# Zika virus sequence and secondary structure analysis using VADR

**DOI:** 10.64898/2026.09.21.753218

**Published:** 2026-09-22

**Authors:** Emma B. Dickinson, Eric P. Nawrocki

## Abstract

Zika virus is a mosquito-borne flavivirus that caused a major epidemic in the Americas in 2015 and 2016 and is associated with congenital birth defects and neurological disease. Like other flaviviruses, it depends on conserved sequence and RNA structural elements that carry out essential steps in its life cycle and in evading host immune defenses. Public sequence databases and annotation tools give free access to Zika sequence data, but comparable structural information is sparse. We built a computational model of Zika sequence and secondary structure for use with the VADR software package, which validates and annotates Zika sequences for GenBank submission and assigns each sequence to a genetic lineage following a published classification scheme. On a test set of 1254 Zika sequences, 97.2% passed validation, and the model’s annotations agreed with those of an existing annotation tool (VIGOR4) for 99.3% of the sequences compared. We also extended VADR to draw secondary structure diagrams of each sequence’s structural elements in its linear and circular conformations using R2DT, and we submitted a newly characterized Zika pseudoknotted RNA element to the Rfam database. Together these freely available resources provide secondary structure information for every Zika sequence.

## Full Text Availability

The license terms selected by the author(s) for this preprint version do not permit archiving in PMC. The full text is available from the preprint server.

